# Role of Sociodemographic Variables and the Mother's Active Behavior on Active Commuting to School in Children and Adolescents

**DOI:** 10.3389/fped.2022.812673

**Published:** 2022-04-04

**Authors:** Fernando Rodriguez-Rodriguez, Patricio Solis-Urra, Jorge Mota, Maria Jesus Aranda-Balboa, Yaira Barranco-Ruiz, Palma Chillon

**Affiliations:** ^1^IRyS Group, School of Physical Education, Pontificia Universidad Católica de Valparaíso, Valparaiso, Chile; ^2^PROFITH “PROmoting FITness and Health Through Physical Activity” Research Group, Department of Physical Education and Sport, Faculty of Sport Sciences, University of Granada, Granada, Spain; ^3^Nuclear Medicine Services, “Virgen de las Nieves” University Hospital, Granada, Spain; ^4^Faculty of Education and Social Sciences, Universidad Andrés Bello, Viña del Mar, Chile; ^5^Faculty of Sport Sciences, Health and Leisure, Research Centre of Physical Activity, University of Porto, Porto, Portugal

**Keywords:** active transport, physical activity, youth, parents, school

## Abstract

The main objective of the current study was to analyze how parents' sociodemographic characteristics, mode of commuting and physical activity (PA) act as indicators of active commuting to school (ACS) in their children and adolescents. A total of 684 paired parents (52.8% mothers) and their respective offspring (33.7% girls) were included. The participants self-reported their sociodemographic characteristics, mode of commuting, and PA. Logistic regression analyses were performed using a stepwise approach, including, as indicators, parental characteristics, mode of commuting and PA. The main outcome was child and adolescent ACS. The odds ratio (OR) and R^2^ of Nagelkerke were obtained for each step. Parental sociodemographic characteristics were greater indicators of child ACS than the parental mode of commuting and PA. In children, the greatest predictive variables of ACS explained 38% of the variance and were as follows: car availability (OR = 0.24), father's educational level (OR = 0.47), mother's educational level (OR = 1.95), mother's active commuting to work (OR = 4.52) and mother's salary/month (OR = 0.67). In adolescents, the greatest predictive variables of ACS explained 40% of the variance and were as follows: socioeconomic level (OR = 0.43) and father's active commuting (OR = 10.6). In conclusion, sociodemographic factors are better indicators of ACS than parents' physical activity and active commuting to work.

## Introduction

Physical activity (PA) in young people has been associated with numerous physical and psychosocial health benefits (1, 2). To obtain these benefits in children and adolescents, the World Health Organization – WHO (3) recommended performing at least 60 daily minutes of moderate-to-vigorous physical activity (MVPA). However, only a small proportion of children and adolescents meet these daily MVPA recommendations (4–6).

On the other hand, the socioecological model has been broadly used to explain the determinants of physical activity (7). This model includes family and people close to the family as highly influential factors for developing these behaviors (8). Accordingly, it has been shown that children's PA habits are associated with their parents' physical activity, especially in younger children (9). Children with active parents are more active than children with inactive parents (10). For instance, a positive association has been found between mother's sport participation and children's out-of-school PA (11). Mothers play a greater role in planning and organizing children's PA, while fathers are more likely to model children's PA (12). According to the previous evidence, the family, and especially, the mothers play an important role in maintaining levels of PA in children, which are worth studying. The educational style of parents and its influence on PA has also been studied. Evidence has found that permissive mothers are associated with higher PA practice by their children than authoritarian mothers (13, 14). In addition, higher levels of care and personalized education for the mother favored athletes and PA practitioners who perceived better support from her parents (15).

Moreover, previous research has demonstrated that active commuting to/from school (ACS), such as walking or cycling, is an opportunity to reach daily PA levels (16–18). Additionally, ACS has been proposed as a strategy where boys and girls are 42% and 66% more likely to attain PA recommendations, respectively (19). However, many children and adults commute to school and work, respectively, using passive modes (20, 21). Furthermore, a general decline in ACS has been observed in recent decades in different countries (22–24) and even in Spain (25). The decline in ACS is partially explained by rising commuting distances and car ownership (26), but other factors may be influences, such as safe routes to school and policies that encourage schools to be placed within neighborhoods to ensure an acceptable walking distance (27). In this regard, different family factors, such as the parent's educational level (15), socioeconomic status (28) and professional levels (29), may influence ACS. However, it is not clearly defined which family factors “are more powerful” to influence this type of active behavior.

In recent years, several studies focused on the established parent-child relationship on ACS have emphasized parental barriers to ACS (30–32). The findings have indicated that parents perceived more barriers in children than adolescents (30, 31, 33). However, few studies have analyzed the influence of parental sociodemographic factors on ACS in offspring (29, 34, 35), which has already been mentioned as relevant. Unfortunately, the studies including in these associations the effect of gender (mothers vs. fathers; boys vs. girls) and including both children and adolescents are lacking. Nevertheless, in the current study the gender variable is included to answer the question about a greater influence of the father or mother. Additionally, fewer studies have investigated the interaction between the active commuting to work (ACW) of parents and the active commuting to/from school (ACS) of their offspring. Recently, Brand et al. (36) found associations between mothers and their children, and Rodríguez-Rodríguez et al. (37) showed a stronger association with children than with adolescents. However, more studies in this regard are needed to elucidate the parental variables and interactions that affect ACS in both children and adolescents.

According to the last, the hypothesis of this study suggests that active behaviors such as PA and ACW have a greater influence on ACS in children and adolescents. At the same time, the influence of fathers and mothers would be different. In this way, understanding how parental factors interact with the ACS of their offspring could offer us key information about how to guide school interventions to achieve successful results, increasing the PA in children and adolescents. Consequently, according to the previous information, the main objective of the current study was to analyze how parental sociodemographic characteristics, mode of commuting, and PA predict ACS in their children and adolescents, highlighting the differences by gender of parents.

## Materials and Methods

### Study Design and Participants

This is a cross-sectional study of schoolchildren and parental participation that was carried out in Granada (Spain) and Valparaíso (Chile). Data were obtained as part of the “Cycling and Walk to School” (PACO, for its Spanish acronym) study, focused on promoting PA and, particularly, active commuting to and from school. The sampling has been obtained for convenience, where initially a total of 2,526 children and adolescents and 1,959 of their parents were invited to participate in the study. Participants came from 20 schools as a nonrandomized sample. From the total sample of children, 1,807 participants could not be paired with parental data, and 34 did not report their gender and were excluded (72.8% of the sample). A total of 572 parents could not be paired with their children's data, and 703 parents without gender data were excluded (65% of the sample). Finally, a total of 684 paired parents (52.8% mothers) and their respective offspring (33.7% girls) were included. These child-parent pairs belonged to 15 schools in Granada, Spain (*n* = 492) and five schools in Valparaíso, Chile (*n* = 192). The age (mean ± standard deviation) of each group was as follows: parents 43.4 ± 6.5 years old, children 9.7 ± 1.7 years old, and adolescents 14.0 ± 1.7 years old.

### Procedures

The student questionnaire was administered to children and adolescents between 2015 and 2018 by the research staff during physical education lessons and lasted ~30 min. The research staff and schoolteachers were present to answer the students' questions (offspring). The parent's questionnaire was delivered to students and completed at home by their parents (mother/father or guardian). Additionally, the parents signed an informed consent form that explained the aims and characteristics of the study and allowed their offspring to participate. The ethical principles for medical research involving human subjects were followed based on The World Medical Association Declaration of Helsinki 2002 revised in 2013. Additionally, the study was reviewed and accepted by the Ethical Committee of the University of Granada, Spain (No. 162/CEIH/2016), and the Ethical Committee of the Pontificia Universidad Católica de Valparaíso, Chile (CCF02052017) to be applied in both countries.

### Parental Sociodemographic Characteristics

The participants self-reported their sociodemographic characteristics, including age, school grade, gender, and full postal address. Additionally, the parent's questionnaire included questions about the highest educational level attained (no study, primary school, secondary school, bachelor's degree, professional degree, and university degree) and income per month (None; <499€; 500–999€; 1,000–1,499€; 1,500–1,999€; 2,000–2,499€; 2,500–2,999€; 3,000–4,999€; >5,000€) dichotomized in <1,000 € and ≥1,000 € according to the minimum salary in Spain which is around 1,000 euros and it has been homogenized for the Chilean sample, where the sample was dichotomized according to the minimum salary in Chile (≅ $350.000). The socioeconomic level was asked using the Family Affluence Scale (FAS) defined with the following questions: “Does your family own a car?” (No [0 point]; Yes, one [1 point]; Yes, two or more [2 points]), “How many computers does your family own?” (None [0 point]; One [1 point]; Two [2 points]; More than two [3 points]), “Do you have your own bedroom for yourself?” (No [0 point]; Yes [1 point]) and “Do you have internet access?” (No [0 point]; Yes [1 point]). A score was assigned for each answer and then summed to obtain the total points. Participants were classified into three categories regarding the FAS: low (0–3 points), medium (4–5 points) and high (6–7 points) (38). In addition, car availability in the family from the FAS was reported as an independent variable.

### Parent's Active Commuting to Work and Physical Activity

To determine the parental mode of commuting to work, two reliable questions (39) were used: (1) “How do you usually get to work?” and (2) “How do you usually get home from work?” The response options for all the questions were walk, bike, car, motorcycle, public bus, metro/train, and other. The usual mode of commuting was categorized as “active” when the parents went to or from to work in “active” mode (walk or bike) and “passive” when the parents went to work in motorized modes (car, motorcycle, public bus, and metro/train). The answer, “Other mode,” was excluded since it was not able to be categorized. In addition, the distance from home to work was asked with the following question: “How far do you live from work? The answer options were <0.5 km, 0.5 to <1 km, 1 to <2 km, 2 to <3 km, 3 to <5 km and >5 km.

The International Physical Activity Questionnaire (IPAQ, short version) was used to determine parental PA levels. The IPAQ shows acceptable psychometric properties to measure MVPA levels in 1 week (40, 41). Additionally, this instrument determines the time in different intensity categories as sedentary, light PA, moderate PA, and vigorous PA in minutes in the last seven days. Regarding MVPA recommendations for adults (≥ 150 min/week), parents were classified as meeting MVPA recommendations (i.e., physically active) and not meeting MVPA recommendations (i.e., physically inactive) (3).

### Active Commuting to School

The children's questionnaire, which included the mode and frequency of commuting to and from school information, was self-reported by the children and adolescents, and has been previously validated (42) and is considered reliable (43) for use in Spanish-speaking children and adolescents. This questionnaire is a 4-item self-report instrument designed to evaluate the mode and weekly frequency of ACS in children and adolescents. Four questions were included in the questionnaire: (1) “How do you usually get to school?”; (2) “How do you usually get home from school?”; (3) “How did you get to school each day?”; and (4) “How did you get home from school each day?”; and the choice of answers to the questions was as follows: walk, bike, car, motorcycle, school bus, public bus, metro/train/tram and other (44). The whole questionnaire is available at http://profith.ugr.es/pages/investigacion/recursos. The mode of commuting was categorized as “active” (walk and bike) or “passive” (car, motorcycle, school bus, public bus, or metro/train/tram). The answer “Other mode” was excluded since it was not able to be categorized. The final dependent variable to predict was the usual active modes of commuting to and from school.

### Statistical Analysis

The mean and standard deviation (SD) for continuous variables and absolute and relative frequency (%) for categorical variables were calculated. The differences in sociodemographic characteristics, mode of commuting to work and PA between mothers and fathers, and the differences in ACS between boys and girls in children and adolescents were calculated using the chi-square test. The results were analyzed separately by country, but not all were calculated due to lack of data. Nor did it show any differences when joining both countries. Therefore, the indicators were calculated from a single group (Chile-Spain together).

Logistic regression analyses were performed stepwise along with three models, including the ACS (passive vs. active) of the children and adolescents as the main outcome variable (passive commuting was used as a reference). The three models were performed separately for children and for adolescents. In the first model, all sociodemographic variables were included as explanatory variables. In the second model, parents' active commuting to work and PA were included as explanatory variables. In the third model, the significant variables from the first two individual models were included as new explanatory variables (all variables) to determine the variables explaining ACS. The odds ratio (OR) and 95% confidence interval of each variable as well as the correlation (Nagelkerke R^2^) were obtained for each step. The confidence interval (CI) values were used to establish the association with ACS. All analyses were performed using SPSS® v21 (IBM, New York, NY, USA). Additionally, a *p* < 0.05 value was considered significant.

## Results

Parental sociodemographic characteristics, mode of commuting to work and PA variables are presented in Table 1. Gender differences were found for educational level, monthly salary, car availability and reaching the MVPA recommendations (mothers: 70.1% and fathers: 51.0%; *p* < 0.001). No significant differences in socioeconomic level or mode of commuting were found between mothers and fathers.

**Table 1:** Parental sociodemographic characteristics, mode of commuting and physical activity variables, overall, and for mothers and fathers.

	**Overall**		**Mothers**		**Fathers**		***p-value***
	***N***	**(%)**	***N***	**(%)**	***N***	**(%)**	
Participants	684 (100)		361 (52.8)		323 (47.2)		
Age (Mean ± SD)	43.4 ± 6.5		42.7 ± 6.5		45.7 ± 6.0		0.094
Educational level (*N* = 631)							
No studies	9	(1.4)	5	(1.6)	4	(1.3)	^b^ <0.001
Primary school	46	(7.3)	32	(10.2)	14	(4.4)	
Secondary school	156	(24.7)	70	(22.3)	86	(27.1)	
Bachelor's	131	(20.8)	47	(22.6)	84	(26.5)	
Professional	122	(19.3)	71	(19.9)	51	(16.1)	
University degree	167	(26.5)	89	(28.3)	78	(24.6)	
Salary/month (*N* = 419)							
Unemployed	33	(7.9)	14	(6.4)	19	(9.5)	
<1,000 €	160	(38.2)	60	(27.3)	100	(50.2)	
1,000 to <2,000 €	182	(43.4)	123	(56.0)	59	(29.6)	^a^ 0.001
2,000 to <3,000 €	39	(9.3)	18	(8.2)	21	(10.6)	
≥3.000 €	5	(1.2)	5	(2.3)	0	(0.0)	
Car availability (*N* = 575)							
None	123	(21.4)	36	(11.5)	87	(33.3)	
Only one	290	(50.4)	167	(53.2)	123	(47.1)	^b^ <0.001
Two or more	162	(28.2)	111	(35.4)	51	(19.6)	
Socioeconomic level (*N* = 381)							
FAS Score (Mean ± DS)	7.26 ± 1.09		7.25 ± 1.11		7.32 ± 1.03		0.608
Mode of commuting to work (*N* = 419)							
Active commuting	82	(20.9)	51	(26.3)	31	(15.6)	0.074
Passive commuting	310	(70.1)	143	(73.7)	167	(84.4)	
MVPA Recommendation (*N* = 519)							
<150 min in MVPA	195	(37.6)	93	(29.9)	102	(49.0)	^b^ <0.001
≥150 min in MVPA	324	(62.4)	218	(70.1)	106	(51.0)	^b^ <0.001

*MVPA, moderate-vigorous physical activity;, SD, standard deviation;*

a *p < 0.05;*

b *p < 0.001*.

The children's and adolescents' sociodemographic characteristics and mode of commuting to school are shown in Table 2. No significant differences between children and adolescents were found, except for age.

**Table 2:** Sociodemographic characteristic and mode of commuting to school between children and adolescents.

	**Overall**		**Children**		**Adolescents**		
	**(*****n*** **= 684)**		**(*****n*** **= 438)**		**(*****n*** **= 246)**		***p-value***
**Sociodemographic factors**	***N***	**(%)**	***N***	**(%)**	***N***	**(%)**	
Age (Mean ± SD)	11.3 ± 2.7		9.7 ± 1.7		14.0 ± 1.7		^a^ <0.001
Gender							
Girls	386	(56.4)	243	(55.5)	143	(58.1)	0.521
Boys	298	(43.6)	195	(44.5)	103	(41.9)	
Mode of commuting (*n* = 673)							
Active	263	(39.1)	169	(39.0)	94	(39.2)	0.518
Passive	410	(60.9)	264	(61.0)	146	(60.8)	

*SD, standard deviation;*

a *p < 0.001*.

The parental sociodemographic characteristics (Model 1) as explanatory variables of ACS in children and adolescents are shown in Table 3.

**Table 3:** Associations between parental sociodemographic characteristics and their child's or adolescent's ACS (Model 1).

**Group**	**Steps**	**Predictors**	***OR***	**CI 95%**	***p*** **-value**	**R^**2**^**
Children	1	Car availability	0.248	0.126–0.489	<0.001	0.18
	2	Car availability	0.282	0.141–0.566	<0.001	0.23
		Father's educational level	0.682	0.507–0.918	0.012	
	3	Car availability	0.290	0.144–0.583	0.001	
		Father's educational level	0.617	0.449–0.847	0.003	0.28
		Age	1.86	1.011–1.166	0.024	
	4	Car availability	0.113	0.037–0347	<0.001	0.32
		Father's educational level	0.571	0.407–0.802	0.001	
		Age	1.088	1.012–1.169	0.022	
		Socioeconomic level	1.894	1.088–3.298	0.024	
Adolescents	1	Socioeconomic level	0.534	0.309–0.924	0.025	0.14

*OR, odds ratio; CI, confidence interval; R^2^, Nagelkerke correlation*.

*Parent variables included in the model were as follows: age, educational level (mother and father), salary/month (mother and father), car availability, and socioeconomic level*.

Regarding children, four-step logistic regressions were obtained in Model 1 of parental sociodemographic characteristics (see **Figure 2**). In the first step, “car availability” was included and explained 18% of the variance; in the second step, “father‘s educational level” was added and explained 23% of the variance (+5%); in the third step, “age” was added, increasing the explained variance to 28% (+5%); and in the last step, “socioeconomic level” was included, increasing the explained variance to 32% (+4%). In this last model, an OR = 1.894 was obtained, increasing the probability that children are active.

In adolescents, only one step was obtained, which included socioeconomic level (OR = 0.534), which explained 14% of the variance.

Model 2 of parental mode of commuting to work and PA variables as explanatory variables of ACS in children and adolescents are shown in Table 4.

**Table 4:** Associations between parental mode of commuting to work and PA with their child's or adolescent's ACS (Model 2).

**Group**	**Steps**	**Predictors**	***OR***	**CI 95%**	***p*** **-value**	**R^**2**^**
Children	1	Father Active Commuting	4.430	2.258–8.691	<0.001	0.09
	2	Father Active Commuting	3.672	1.826–7.381	<0.001	0.14
		Mother Active Commuting	3.363	1.580–7.161	0.002	
	3	Father Active Commuting	4.269	2.064–8.828	<0.001	
		Mother Active Commuting	3.247	1.509–6.987	0.003	0.16
		Mother >150 min in MVPA	1.961	1.079–3.563	0.027	
Adolescents	1	Father Active Commuting	3.142	1.108–8.913	0.031	0.05

*OR, odds ratio; CI, confidence interval; R^2^, Nagelkerke correlation*.

*Included parent variables in the model: MVPA (mother and father) and mode of commuting (mother and father)*.

In children, three steps were calculated to predict ACS (see **Figure 2**). The first step included a higher “father ACW,” explaining only 9% of the variance, but a greater odds value (OR = 4.430). As a second step, lesser “mother ACW” was included in the model, increasing the variance explanation by 14% (+5%). The third step included a higher “mother ≥150 minutes in MVPA,” slightly increasing the variance explanation toward 16% (+2%). In adolescents, only one step was used to predict ACS. Higher “Father ACW” explained 5% of the variance.

**Figure 1:**
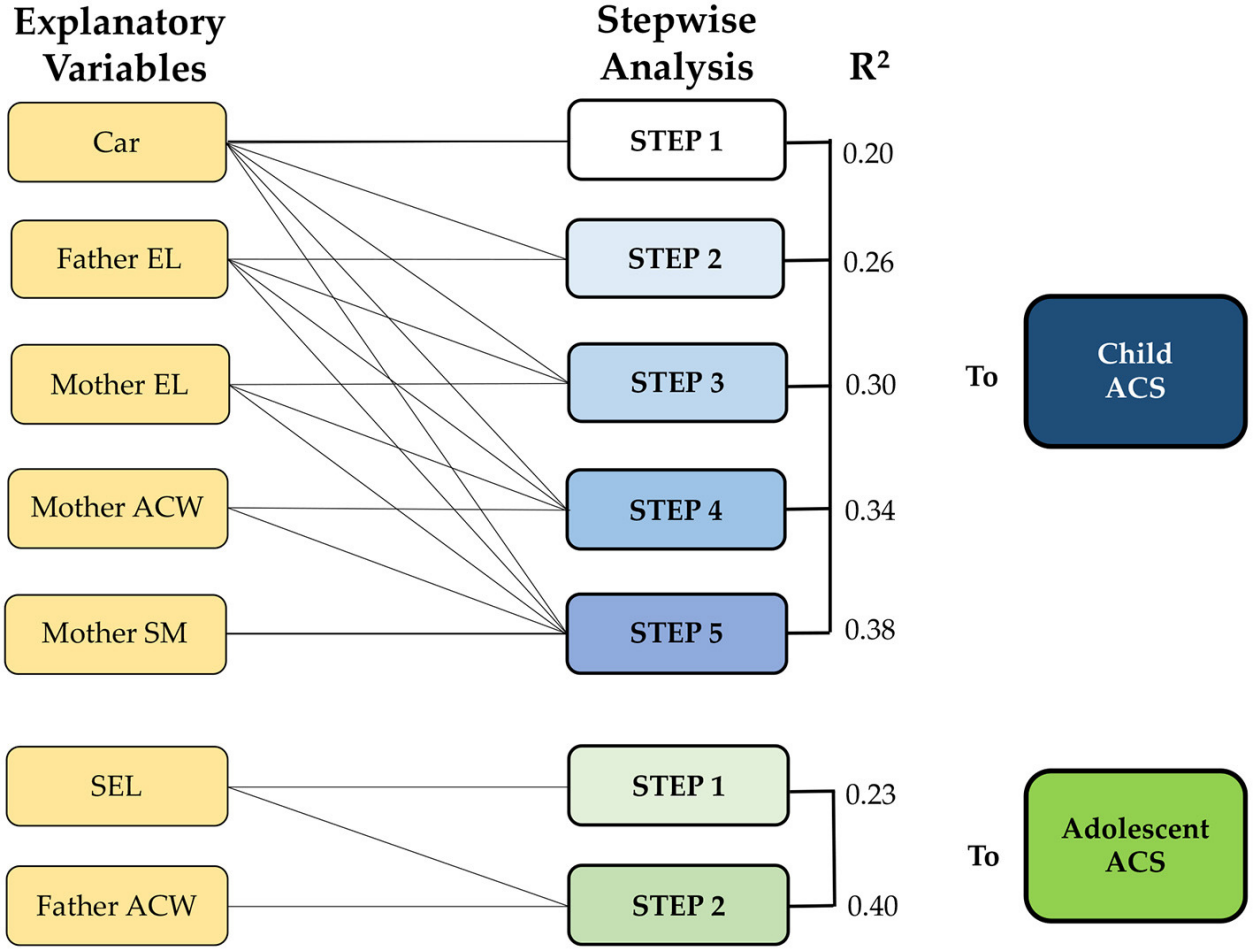
Nagelkerke correlation (R^2^) in combined stepwise model analysis (Model 3) on active commuting to school in children and adolescents. Car, car availability; EL, educational level; ACW, active commuting to work; SM, salary per month; SEL, socioeconomic level; ACS, active commuting to school.

**Figure 2:**
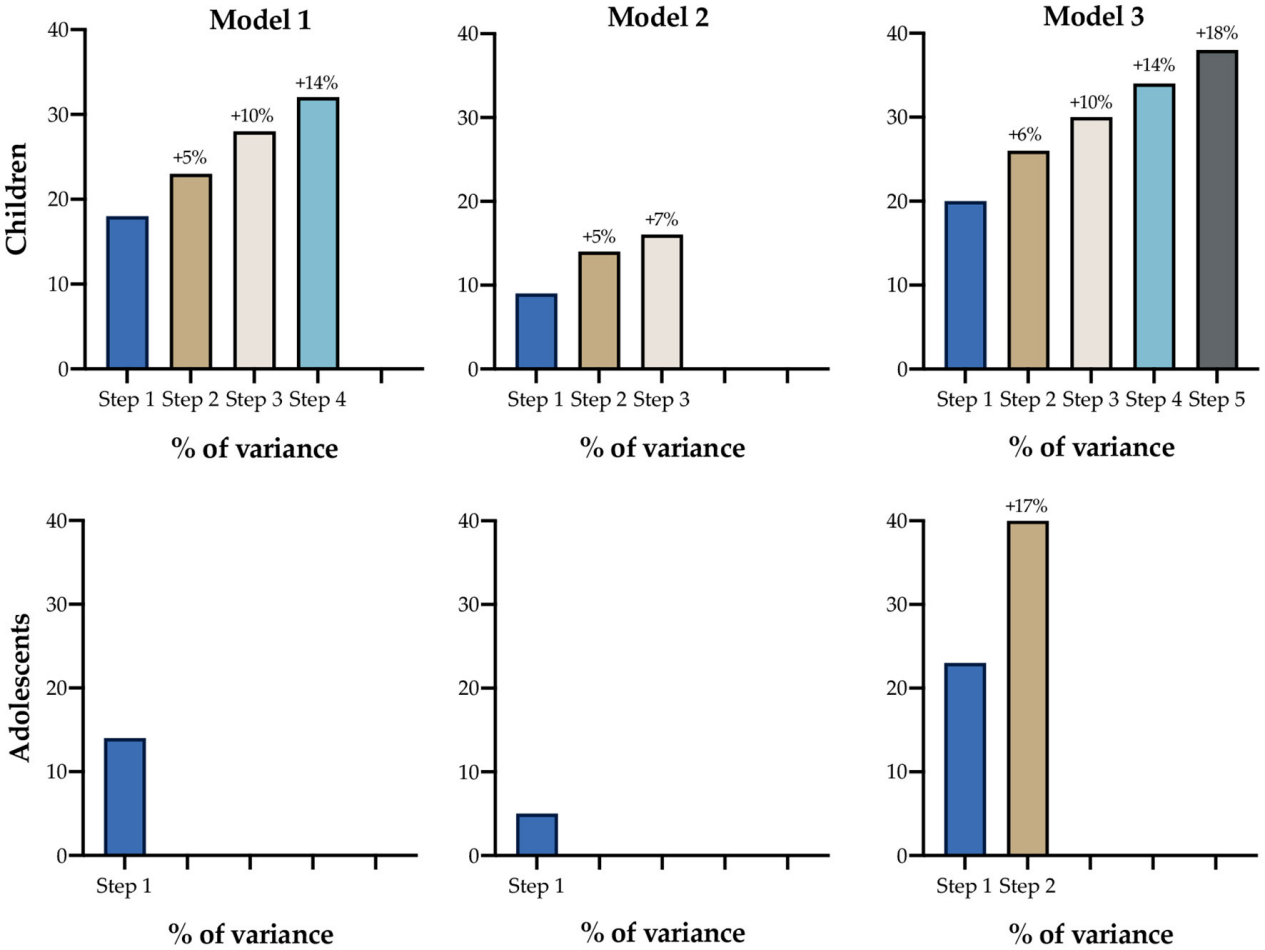
Increase in the percentage of variance explained in each model in children and adolescents.

Every previous parental explanatory variable was included in Model 3, separately, for children and adolescents (Figure 1), and higher variance values were obtained. Regarding children, five stepwise models, which included “car availability,” less “father's educational level” (+6%), higher “mother's educational level” (+4%), “mother ACW” (+4%) and less mother salary/month (+4%) as explanatory variables of ACS (see Figure 2). Overall, the model explained 38% of the variance.

Regarding adolescents, two steps were calculated to predict ACS. The first step included less “socioeconomic level” and explained 23% of the variance, while the second step included the higher “Father ACW” (+17%) and increased the variance explanation toward 40%.

Figure 2 shows the differences between the three models stratified by children and adolescents. In addition, the increases in the percentage of variance explained can be seen when adding each variable (steps).

## Discussion

### Physical Activity and Commuting to Work of Parents

The main objective of the current study was to analyze how parental sociodemographic characteristics, mode of commuting, and PA predict ACS in their children and adolescents, highlighting the differences by gender of parents. Moreover, the main results of the current study were that parental sociodemographic characteristics, such as car availability, mother's and father's educational levels, mother's salary, and socioeconomic level, explained significantly more the ACS than ACW and parent' PA. In accordance with this, the hypothesis is rejected, which stated that active behaviors such as PA and ACW have a greater influence on ACS in children and adolescents.

According to the above, only the mother's PA was incorporated into the Model 2 to explain ACS in children. But it only improved the variance from 14 to 16%. An important implication of these findings is that the weak association between parental PA and ACS can be explained because the questionnaire (IPAQ) applied in the parents tends to overestimate the amount of physical activity reported compared to an objective device (45). In this way, any association could be lost and, consequently, these results must be analyzed with caution. In addition, this could be explained by the low number of parents who met the PA recommendations (37).

Parental behavior affects children's behavior, and the importance of the family in the development of children's active behaviors has been previously demonstrated (46). Regarding active commuting to work, mothers' active commuting to work in children and fathers' active commuting to work in adolescents were important variables for explaining ACS. Recently, a study conducted in North America with 344 parents suggested that ACS among children was directly influenced by the commuting behaviors of their parents (10). In another study in Brazil, strong associations between children's and adolescents' ACS with the mother's active commuting to work were found (36). Additionally, greater positive associations were found for mothers actively commuting to work and their offspring's ACS and stronger associations between parents and children than adolescents were found (37). To our knowledge, few studies have associated the active commuting of parents with children with ACS, and future studies should continue to explore and to describe new relationships.

### Sociodemographic

Model 3, which included the sociodemographic variables, active commuting, and PA, was the more efficient explanatory model. In the case of children, four of the five variables that best predicted ACS were sociodemographic factors (car availability, father's educational level, mother's educational level and mother's salary/month).

Car availability usually represents a high socioeconomic level and has regularly been associated with lower ACS (29, 44, 47, 48). In addition, families without a car increase their active commuting options (49). However, parents can conveniently drive to school because trips can often be combined with work commutes (50). Additionally, a long distance to commuting home to school or home to work typically involves car use (51), and thus negatively affects ACS. A study conducted in New Zealand showed that 32.3% of schoolchildren enrolled in the closest school were driven to school by car, compared to 57.2% not enrolled in the closest school that were driven by car (52).

In another sense, our findings demonstrated that children's ACS can be explained by the educational levels of fathers and mothers. A previous study showed that parents with lower levels of education drove cars less to work (38.8 vs. 46.6%), and they walked more often (19.1 vs. 16.9%) than parents with high educational levels (21).

In adolescents, parental educational level was not an explanatory variable for ACS. However, socioeconomic level was an important sociodemographic variable included in the model. A Spanish study showed that adolescents from families with high socioeconomic levels had lower levels of ACS than their peers from families with low socioeconomic levels (44). It should be considered that sociodemographic variables are less modifiable and cannot be intervened. Therefore, interventions must identify the factors that may influence the increase or decrease in ACS. Therefore, our results indicate that parental and familial sociodemographic factors have the most important role in the ACS of children and adolescents.

Our study has described several parental factors that can explain ACS and help to focus future intervention strategies.

## Strengths and Limitations

The large sample of parents and their offspring stand out as one of the strengths of the study, reaching 1,368 participants. Data from two Spanish-speaking countries were enrolled with their respective language adaptations, providing instruments that can be used by other Latin American countries. Additionally, the novelty of the study was to have included sociodemographic and PA variables in the same model, which provided new evidence on parents and their offspring.

The main limitation of the study was the cross-sectional design; therefore, no cause-and-effect relationship can be established in the associations. Indeed, a longitudinal study is required to determine the causal direction of the relationship. There was a relevant loss of sample data regarding the initial data collection because there were many incomplete questionnaires. Additionally, a nonrandomized sample was included; therefore, it is not possible to generalize to other populations. In addition, a self-reported questionnaire was used, which has a lower objectivity to determine PA than devices such as accelerometers.

## Conclusion

The most explanatory variables for children's ACS, ordered from more to less relevance, were the parents' car availability, father's educational level, mother's educational level, mother's salary/month and mother's active commuting to work. The most explanatory variables for adolescents' ACS, ordered from more to less relevance, were socioeconomic level and father's active commuting. In conclusion and according to our objective, it can be stated that parental sociodemographic factors are more related to active commuting to school in children and adolescents than parents'physical activity and active commuting to work. In addition, more factors from the mother influence this active behavior.

## Data Availability Statement

The raw data supporting the conclusions of this article will be made available by the authors, without undue reservation.

## Ethics Statement

The studies involving human participants were reviewed and approved by Bioethical Committee of the Pontificia Universidad Católica de Valparaíso, Chile (CCF02052017). Written informed consent to participate in this study was provided by the participants' legal guardian/next of kin.

## Author Contributions

FR-R, PS-U, JM, MA-B, YB-R, and PC have made substantial contributions to the conception of the study and drafted the work and substantively revised it. FR-R and PS-U in design of the work and analysis of data. MA-B in acquisition of data. FR-R, PS-U, JM, and PC interpretation of data. All authors contributed to the article and approved the submitted version.

## Funding

This study was supported by Spanish Ministry of Economy, Industry and Competitiveness and European Regional Development Fund. Additionally, this study took place thanks to funding from University of Granada Plan Propio de Investigación 2016—Excellence actions: Unit of Excellence on Exercise and Health (UCEES)—and Junta de Andalucía, Consejería de Conocimiento, Investigación y Universidades, and European Regional Development Fund (ref. SOMM17/6107/UGR). Additionally, this work was supported by Ministry of Education of Chile CONICYT PAI-MEC programme 2015 (MEC 80150030) and the Postdoctoral programme Becas Chile 2019 from Agencia Nacional de Investigación y Desarrollo de Chile (ANID).

## Conflict of Interest

The authors declare that the research was conducted in the absence of any commercial or financial relationships that could be construed as a potential conflict of interest.

## Publisher's Note

All claims expressed in this article are solely those of the authors and do not necessarily represent those of their affiliated organizations, or those of the publisher, the editors and the reviewers. Any product that may be evaluated in this article, or claim that may be made by its manufacturer, is not guaranteed or endorsed by the publisher.

## References

[1] Poitras VJ Gray CE Borghese MM Carson V Chaput JP Janssen I. Systematic review of the relationships between objectively measured physical activity and health indicators in school-aged children and youth. Appl Physiol Nutr Metab. (2016) 41:S197–239. 10.1139/apnm-2015-0663 27306431

[2] Rhodes RE Janssen I Bredin SS Warburton DE Bauman A. Physical activity: Health impact, prevalence, correlates and interventions. Psychol Health. (2017) 32:942–75. 10.1080/08870446.2017.1325486 28554222

[3] Bull FC Al-Ansari SS Biddle S Borodulin K Buman MP Cardon G. World Health Organization 2020 guidelines on physical activity and sedentary behaviour. Brit J Sports Med. (2020) 54:1451–62. 10.1136/bjsports-2020-102955 33239350 PMC7719906

[4] Hallal PC Andersen LB Bull FC Guthold R Haskell W Ekelund U. Lancet physical activity series working group. global physical activity levels: surveillance progress, pitfalls, and prospects. Lancet. (2012) 380:247–57. 10.1016/S0140-6736(12)60646-1 22818937

[5] Guthold R Stevens GA Riley LM Bull FC. Global trends in insufficient physical activity among adolescents: a pooled analysis of 298 population-based surveys with 1¬∑ 6 million participants. Lancet Child Adolesc Health. (2020) 4:23–35. 10.1016/S2352-4642(19)30323-2 31761562 PMC6919336

[6] Vancampfort D Van Damme T Firth J Smith L Stubbs B Rosenbaum S. Correlates of physical activity among 142,118 adolescents aged 12–15 years from 48 low-and middle-income countries. Prev Med. (2019) 127:105819. 10.1016/j.ypmed.2019.105819 31445918

[7] Sallis JF Cervero RB Ascher W Henderson KA Kraft MK Kerr J. An Ecological Approach To Creating Active Living Communities. Annu Rev Public Health. (2006) 27:297–322. 10.1146/annurev.publhealth.27.021405.102100 16533119

[8] Mehtälä MAK Sääkslahti AK Inkinen ME Poskiparta MEH. A socio-ecological approach to physical activity interventions in childcare: a systematic review. Int J Behav Nutr Phys Act. (2014) 11:1–12. 10.1186/1479-5868-11-22 24559188 PMC3936868

[9] Ha AS Ng JYY Lonsdale C Lubans DR Ng FF. Promoting physical activity in children through family-based intervention: protocol of the “Active 1+FUN” randomized controlled trial. BMC Public Health. (2019) 19:218. 10.1186/s12889-019-6537-3 30786902 PMC6383281

[10] Sims D Bopp M. Using parental active travel behavior and beliefs to predict active travel to school among children. Int J Sustain Transp. (2019) 1–6. 10.1080/15568318.2018.1558469

[11] Arlinghaus KR Johnston CA. Engaging fathers in the promotion of healthy lifestyle behaviors. AJLM. (2017) 11:216–9. 10.1177/1559827617690724 30202333 PMC6125089

[12] Lloyd AB Lubans DR Plotnikoff RC Collins CD Morgan PJ. Maternal and paternal parenting practices and their influence on children's adiposity, screen-time, diet and physical activity. Appetite. (2014) 79:149–57. 10.1016/j.appet.2014.04.010 24751915

[13] Hennessy E Hughes SO Goldberg JP Hyatt RR Economos CD. Parent–child interactions and objectively measured child physical activity: a cross-sectional study. Int J Behav Nutr Phys Act. (2010) 7:1–14. 10.1186/1479-5868-7-71 20929570 PMC2964559

[14] Jago R Davison KK Brockman R Page AS Thompson JL Fox KR. Parenting styles, parenting practices, and physical activity in 10 to 11 year olds. Prev Med. (2011) 52:44–7. 10.1016/j.ypmed.2010.11.001 21070805 PMC3025352

[15] González-García H Muñoz AP Crespo JLC. Protective Parents, Democratic Parents and Support to Physical Activity and Sport. Cult Cienc Deporte. (2019) 14:51–9. 10.12800/ccd.v14i40.1225

[16] Martin A Kelly P Boyle J Corlett F Reilly JJ. Contribution of walking to school to individual and population moderate-vigorous intensity physical activity: systematic review and meta-analysis. Pediatr Exerc Sci. (2016) 28:353–63. 10.1123/pes.2015-0207 26882871

[17] Prince SA Butler GP Rao DP Thompson W. Evidence synthesis Where are children and adults physically active and sedentary?–a rapid review of location-based studies. Health Promot Chronic Dis Prev Can. (2019) 39:67. 10.24095/hpcdp.39.3.01 30869472 PMC6478053

[18] Chillon P Ortega FB Ruiz JR Veidebaum T Oja L Mäestu J. Active commuting to school in children and adolescents: an opportunity to increase physical activity and fitness. Scand J Public Health. (2010) 38:873–9. 10.1177/1403494810384427 20855356

[19] Peralta M Henriques-Neto D Bordado J Loureiro N Diz S Marques A. Active Commuting to School and Physical Activity Levels among 11 to 16 Year-Old Adolescents from 63 Low- and Middle-Income Countries. Int J Environ Res Public Health. (2020) 17:1276. 10.3390/ijerph17041276 32079217 PMC7068453

[20] Aubert S Barnes JD Abdeta C Abi Nader P Adeniyi AF Aguilar-Farias N. Global matrix 30 physical activity report card grades for children and youth: results and analysis from 49 countries. J Phys Act Health. (2018) 15:S251–73. 10.1123/jpah.2018-0472 30475137

[21] Te Velde SJ Haraldsen E Vik FN De Bourdeaudhuij I Jan N Kovacs E. Associations of commuting to school and work with demographic variables and with weight status in eight European countries: The ENERGY-cross sectional study. Prev Med. (2017) 99:305–12. 10.1016/j.ypmed.2017.03.005 28315759

[22] Costa FF Silva KS Schmoelz CP Campos VC de Assis MAA. Longitudinal and cross-sectional changes in active commuting to school among Brazilian schoolchildren. Prev Med. (2012) 55:212–4. 10.1016/j.ypmed.2012.06.023 22772080

[23] Pavelka J Sigmundová D Hamrík Z Kalman M Sigmund E Mathisen F. Trends in Active Commuting to School among Czech Schoolchildren from 2006 to 2014. Cent Eur J Public Health. (2017) 25(Suppl 1):S21–S25. 10.21101/cejph.a5095 28752743

[24] Reimers AK Marzi SC Schmidt I Niessner C Oriwol D Worth A. Trends in active commuting to school from 2003 to 2017 among children and adolescents from Germany: the MoMo Study. Eur J Public Health. (2020). 10.1093/eurpub/ckaa141 33011779

[25] Chillón P Martínez-Gómez D Ortega FB Pérez-López IJ Díaz LE Veses AM. Six-year trend in active commuting to school in Spanish adolescents. Int J Behav Med. (2013) 20:529–37. 10.1007/s12529-012-9267-9 23055026

[26] McDonald NC. Active transportation to school: trends among US schoolchildren, 1969–2001. Am J Prev Med. (2007) 32:509–16. 10.1016/j.amepre.2007.02.022 17533067

[27] Sener IN Lee RJ Sidharthan R. An examination of children's school travel: a focus on active travel and parental effects. Transp Res A Policy Pract. (2019) 123:24–34. 10.1016/j.tra.2018.05.023

[28] D'Haese S Van Dyck D De Bourdeaudhuij I Deforche B Cardon G. The association between objective walkability, neighborhood socio-economic status, and physical activity in Belgian children. Int J Behav Nutr Phys Act. (2014) 11:104. 10.1186/s12966-014-0104-1 25148724 PMC4243938

[29] Rodríguez-López C Villa-González E Pérez-López IJ Delgado-Fernández M Ruiz JR Chillón P. Family factors influence active commuting to school in Spanish children. Nutr Hosp. (2013) 28:756–63. 23848100 10.3305/nh.2013.28.3.6399

[30] Aranda-Balboa MJ Huertas-Delgado FJ Herrador-Colmenero M Cardon G Chillón P. Parental barriers to active transport to school: a systematic review. Int J Public Health. (2019) 1–12. 10.1007/s00038-019-01313-1 31728600

[31] Palma X Chillón P Rodríguez-Rodríguez F Barranco-Ruiz Y Huertas-Delgado FJ. Perceived parental barriers towards active commuting to school in Chilean children and adolescents of Valparaíso. Int J Sustain Transp. (2019) 1–8. 10.1080/15568318.2019.1578840

[32] Solana AA Mandic S Lanaspa EG Gallardo LO Casterad JZ. Parental barriers to active commuting to school in children: does parental gender matter? J Transp Health. (2018) 9:141–9. 10.1016/j.jth.2018.03.005

[33] Huertas-Delgado FJ Chillón P Barranco-Ruiz Y Herrador-Colmenero M Rodríguez-Rodríguez F Villa-González E. Parental perceived barriers to active commuting to school in Ecuadorian youth. J Transp Health. (2018) 10:290–6. 10.1016/j.jth.2018.05.102

[34] Henne HM Tandon PS Frank LD Saelens BE. Parental factors in children's active transport to school. Public Health. (2014) 128:643–6. 10.1016/j.puhe.2014.05.004 24999161 PMC4149318

[35] Carlson JA Sallis JF Kerr J Conway TL Cain K Frank LD. Built environment characteristics and parent active transportation are associated with active travel to school in youth age 12-15. Br J Sports Med. (2014) 48:1634–9. 10.1136/bjsports-2013-093101 24659503 PMC4447304

[36] Brand C Reuter CP Dias AF Mota J Duncan M Gaya AR. Like Mother, like Son: Physical Activity, Commuting, and Associated Demographic Factors. Sustainability. (2020) 12:5631. 10.3390/su12145631

[37] Rodríguez-Rodríguez F Huertas-Delgado FJ Barranco-Ruiz Y Aranda-Balboa MJ Chillón P. Are the Parents' and Their Children's Physical Activity and Mode of Commuting Associated? Analysis by Gender and Age Group. Int J Environ Res Public Health. (2020) 17:6864. 10.3390/ijerph17186864 32962197 PMC7558568

[38] Boyce W Torsheim T Currie C Zambon A. The family affluence scale as a measure of national wealth: validation of an adolescent self-report measure. Soc Indic Res. (2006) 78:473–87. 10.1007/s11205-005-1607-6

[39] Herrador-Colmenero M Ruiz JR Ortega FB Segura-Jiménez V Álvarez-Gallardo IC Camiletti-Moirón D. Reliability of the ALPHA environmental questionnaire and its association with physical activity in female fibromyalgia patients: The al-Ándalus project. J Sports Sci. (2015) 33:850–62. 10.1080/02640414.2014.968190 25357996

[40] Craig CL Marshall AL Sjöström M Bauman AE Booth ML Ainsworth BE. International physical activity questionnaire: 12-country reliability and validity. Med Sci Sports Exerc. (2003) 35:1381–95. 10.1249/01.MSS.0000078924.61453.FB 12900694

[41] Roman-Viñas B Serra-Majem L Hagströmer M Ribas-Barba L Sjöström M Segura-Cardona R. International physical activity questionnaire: reliability and validity in a Spanish population. Eur J Sport Sci. (2010) 10:297–304. 10.1080/17461390903426667

[42] Chillón P Herrador-Colmenero M Migueles JH Cabanas-Sánchez V Fernández-Santos JR Veiga OL. Convervent validation of a questionnaire to assess the mode and frequency of commuting to and from school. Scand J Public Health. (2017) 45:612–20. 10.1177/1403494817718905 30747037

[43] Segura-Díaz JM Rojas-Jiménez Á Barranco-Ruiz Y Murillo-Pardo B Saucedo-Araujo RG Aranda-Balboa MJ. Feasibility and Reliability of a Questionnaire to Assess the Mode, Frequency, Distance and Time of Commuting to and from School: The PACO Study. Int J Environ Res Public Health. (2020) 17:5039. 10.3390/ijerph17145039 32668796 PMC7399968

[44] Chillón P Ortega FB Ruiz JR Pérez IJ Martín-Matillas M Valtueña J. Socio-economic factors and active commuting to school in urban Spanish adolescents: the AVENA study. Eur J Public Health. (2009) 19:470–6. 10.1093/eurpub/ckp048 19535607

[45] Lee PH Macfarlane DJ Lam TH Stewart SM. Validity of the international physical activity questionnaire short form (IPAQ-SF): a systematic review. Int J Behav Nutr Phys Act. (2011) 8:1–11. 10.1186/1479-5868-8-115 22018588 PMC3214824

[46] Christofaro DG Turi-Lynch BC Lynch KR Tebar WR Fernandes RA Tebar FG. Parents' lifestyle, sedentary behavior, and physical activity in their children: a Cross-sectional study in Brazil. J Phys Act Health. (2019) 16:631–6. 10.1123/jpah.2018-0173 31310992

[47] Molina-García J Queralt A. Neighborhood Built Environment and Socioeconomic Status in Relation to Active Commuting to School in Children. J Phys Act Health. (2017) 14:761–5. 10.1123/jpah.2017-0033 28513318

[48] Molina-García J Menescardi C Estevan I Martínez-Bello V Queralt A. Neighborhood built environment and socioeconomic status are associated with active commuting and sedentary behavior, but not with leisure-time physical activity, in university students. Int J Environ Res Public Health. (2019) 16:3176. 10.3390/ijerph16173176 31480418 PMC6747177

[49] Terrón-Pérez M Molina-García J Martínez-Bello VE Queralt A. Active commuting to school among preschool-aged children and its barriers: an exploratory study in collaboration with parents. J Transp Health. (2018) 8:244–50. 10.1016/j.jth.2017.12.007

[50] McDonald NC Aalborg AE. Why parents drive children to school: implications for safe routes to school programs. J Am Plan Assoc. (2009) 75:331–42. 10.1080/01944360902988794

[51] Döring L Kroesen M Holz-Rau C. The role of parents' mobility behavior for dynamics in car availability and commute mode use. Transportation. (2019) 46:957–94. 10.1007/s11116-017-9823-x

[52] Mandic S Sandretto S Bengoechea EG Hopkins D Moore A Rodda J. Enrolling in the Closest School or Not? Implications of school choice decisions for active transport to school. J Transp Health. (2017) 6:347–57. 10.1016/j.jth.2017.05.006

